# Nanotube‐mediated plasmid transfer as a natural alternative for the improvement of industrially relevant bacteria

**DOI:** 10.1111/1751-7915.14225

**Published:** 2023-02-11

**Authors:** Carlos Molina‐Santiago, Patricia Bernal

**Affiliations:** ^1^ Departamento de Microbiología, Instituto de Hortofruticultura Subtropical y Mediterránea “La Mayora”, Universidad de Málaga – Consejo Superior de Investigaciones Científicas (IHSM‐UMA‐CSIC) Universidad de Málaga Málaga Spain; ^2^ Departamento de Microbiología, Facultad de Biología Universidad de Sevilla Seville Spain

DNA transfer between prokaryotic neighbouring cells is a well‐known phenomenon that occurs widely in nature and has a significant impact on microbial ecology and evolution. The rapid acquisition of new genetic material, that may have new features, allows bacteria to quickly adapt to changing and challenging environments. The classical mechanisms of horizontal gene transfer (HGT) include natural transformation, that is, the uptake of DNA from the environment, conjugation, that is, cell‐to‐cell DNA transfer, and transduction, that is, transmission of genomic DNA via phages. These mechanisms require a special state in recipient cells (competence) or a recognition between recipient and donor cells. In recent years, mostly unexplored cellular structures such as membrane vesicles (MVs) and nanotubes have been proposed to participate in the exchange of cytoplasmic molecules, including DNA, between nearby cells. Investigations into these structures are in the early stages, and limited information is available about the molecular mechanisms governing these organelles or the real implications of these structures in HGT. Membrane vesicles were first discovered in Gram‐negative bacteria, where they were found to originate from controlled blebbing of the outer membrane (Schwechheimer & Kuehn, 2015). Later, similar structures were discovered in Gram‐positive bacteria and different mechanisms of MV formation were described, such as endolysin‐triggered cell lysis (Abe et al., 2021). Nanotubes are also membranous structures, but unlike membrane vesicles, they directly and physically connect adjacent bacterial cells. They were discovered and first characterized in 2011 in the laboratory of Prof. Ben‐Yehuda using *Bacillus subtilis* as a model organism (Dubey & Ben‐Yehuda, 2011). These structures are assembled from conserved components of the flagellar export apparatus, that is, FliOPQR and FlhAB (Bhattacharya et al., 2019), and are composed of chains of lipid bilayer fragments that shape and hold a continuous lumen (Dubey et al., 2016). In this manner, nanotubes bridge contiguous bacteria of the same or different species. This allows the trade of cytoplasmic material, including proteins and DNA, within and between species. In their seminal work, Dubey and Ben‐Yehuda (2011) described the transfer of non‐conjugative plasmids from one *B. subtilis* cell to another via nanotubes. This study also described the formation of nanotubes intraspecies, specifically between *B. subtilis* and *Staphylococcus aureus*, both Gram‐positive bacteria and between *B. subtilis* and the Gram‐negative bacteria *Escherichia coli*. However, the transfer of non‐conjugative plasmids between these species was not tested in this work (Dubey & Ben‐Yehuda, 2011).

Recently, Morawska and Kuipers (2022) demonstrated that non‐conjugative plasmids can be transferred via nanotubes between species such as *Streptococcus thermophilus*, *Lactococcus lactis* and the well‐studied nanotube producer *B. subtilis*. This seems to be an underestimated HGT mechanism, and, based on the widespread occurrence of nanotubes in bacteria, it is likely a universal process. The authors of this work, recently published in Microbial Biotechnology, initially demonstrated the feasibility of the transfer of non‐conjugative plasmids between *B. subtilis* strains. They used a *comK* mutant as a recipient strain to prevent natural competence and utilized antibiotics as markers to select recipient strains carrying the transferred plasmid. The authors also considered the presence of prophages (SPβ) and other integrative and conjugative elements such as ICE*Bs1* codified in the genome of *B. subtilis* 168 (Auchtung et al., 2016; Floccari & Dragoš, 2023), as they can be involved, directly or indirectly, in DNA transfer. Thus, they used a strain cured from these two elements to verify that the plasmid transfer they were visualizing was a cell‐to‐cell contact‐dependent process and not the result of conjugative events or induced prophage transduction (Morawska & Kuipers, 2022).

This remarkable mechanism has been studied in classical laboratory‐model bacteria such as *B. subtilis*. Of greater interest are the promising biotechnological applications that this HGT mechanism could provide as a natural mechanism to improve industrially relevant bacteria. As demonstrated by Morawska and Kuipers, the transfer of plasmids from and to Lactic Acid Bacteria (LAB) is a reality. LAB strains are commonly used in the food industry for the production of dairy products and lack competence genes. Importantly, this work establishes that non‐conjugative plasmid transfer occurs in both directions, from *B. subtilis* to LAB and vice versa, being nanotubes the only structure necessary for DNA exchange. Moreover, the authors determined that this is a widely spread mechanism by demonstrating plasmid transfer between LAB strains in the absence of the lactococcal sex factor; an element necessary for DNA mobilization. Furthermore, they examined the functionality of cell‐to‐cell plasmid transfer between other industrially relevant strains such as *S. thermophilus*, which is used in milk fermentation processes.

The European food industry prohibits the use of genetically modified organisms (GMOs). This limits the development of improved strains by using CRISPR‐Cas approaches or other recombination techniques (Plavec & Berlec, 2020). The intra and interspecies acquisition of DNA by natural means such as the one reported by Morawska and Kuipers (2022) in Microbial Biotechnology is a promising strategy to develop industrial strains.

As with any newly discovered system, many crucial questions still need to be answered such as the universality of the system, the underlying molecular mechanisms (e.g. the DNA size that can be transferred), or the ethical and legal concerns that will arise from its use. Therefore, many facts must be considered before the establishment of this strategy in the industrial sector. However, it is undeniable the importance of this system from both, a biological and an industrial point of view and its great potential for applications in the food and agriculture industries to accomplish their needs by bypassing GMO regulations.

## AUTHOR CONTRIBUTIONS

**Carlos Molina‐Santiago:** Writing – original draft‐Equal, Writing – review & editing‐Equal. **Patricia Bernal:** Writing – original draft‐Equal, Writing – review & editing‐Equal.

## CONFLICT OF INTEREST STATEMENT

The authors declare that they have no conflict of interest.
